# L-DOPA changes spontaneous low-frequency BOLD signal oscillations in Parkinson's disease: a resting state fMRI study

**DOI:** 10.3389/fnsys.2012.00052

**Published:** 2012-07-04

**Authors:** Y. Kwak, S. J. Peltier, N. I. Bohnen, M. L. T. M. Müller, P. Dayalu, R. D. Seidler

**Affiliations:** ^1^Neuroscience Program, University of Michigan, Ann ArborMI, USA; ^2^Department of Biomedical Engineering, University of Michigan, Ann ArborMI, USA; ^3^Department of Radiology, University of Michigan, Ann ArborMI, USA; ^4^Department of Neurology, University of Michigan, Ann ArborMI, USA; ^5^GRECC, VA Ann Arbor Healthcare System, University of Michigan, Ann ArborMI, USA; ^6^School of Kinesiology, University of Michigan, Ann ArborMI, USA; ^7^Department of Psychology, University of Michigan, Ann ArborMI, USA; ^8^Institute of Gerontology, University of Michigan, Ann ArborMI, USA

**Keywords:** Parkinson's disease, resting-state fMRI, dopamine, neural oscillation, BOLD signal

## Abstract

Analysis of the amplitude of low frequency BOLD signal fluctuations (ALFF) in the resting state has recently been used to study the dynamics of intrinsic neural activity. Several studies have also suggested its potential as a biomarker for neuropsychiatric disease. In the current study, we quantified ALFF to determine changes in intrinsic neural oscillations in patients with Parkinson's disease (PD) on and off L-DOPA. Twenty-four PD patients and 24 healthy age-matched controls participated in the study. PD patients underwent two resting state fMRI sessions, either ON a controlled dose of L-DOPA or following a placebo pill (OFF). Control participants underwent one test session. We found that there was increased amplitude of low frequency BOLD signal oscillations for PD patients OFF L-DOPA in the primary and secondary motor areas, and in the middle and medial prefrontal cortices. L-DOPA significantly reduced the amplitude of low frequency oscillations within these regions. The degree of ALFF in the premotor cortex predicted patients' motor performance as measured by the Grooved Pegboard task, such that greater ALFF was associated with poorer performance. These results are in line with the pathophysiology of PD, which shows changes in neural oscillations. Thus, frequency domain analyses of resting state BOLD fMRI signals may provide a useful means to study the pathophysiology of PD and the physiology of the brain's dopaminergic pathways.

## Introduction

Parkinson's disease (PD) is a progressive neurodegenerative disorder associated with loss of dopaminergic neurons in the substantia nigra pars compacta and the ventral tegmental area with degeneration of the striatal nerve terminals (Bernheimer et al., 1973; Kish et al., 1988; Frey et al., 1996; Rakshi et al., 1999; Braak et al., 2006). One consistent pathophysiological hallmark of PD is a change in spontaneous neural oscillations across the corticostriatal networks (Schnitzler and Gross, 2005; Gatev et al., 2006; Berendse and Stam, 2007; Hammond et al., 2007; Timmermann and Fink, 2009). Several studies show changes in low frequency neural oscillations (0.3–2.5 Hz) in rodent models of PD (Magill et al., 2001; Tseng et al., 2001; Belluscio et al., 2003; Walters et al., 2007). These studies have shown elevation of synchronous activation between cortical and basal ganglia neurons (Tseng et al., 2001) or an augmentation of oscillatory activity within basal ganglia nuclei (Magill et al., 2001; Belluscio et al., 2003; Walters et al., 2007) at low frequencies. Parallel results are reported in human patients using EEG or MEG. PD associated changes in EEG signal oscillations are found in a range of frequencies. For example, patients showed a widespread slowing of oscillatory brain activity reflected as an increase in the power of neural activity at the low frequency ranges such as the theta and low alpha bands (Soikkeli et al., 1991; Bosboom et al., 2006; Stoffers et al., 2007; Moran et al., 2008). Other studies show prominent increases in beta band oscillations (Kuhn et al., 2004, 2006; Foffani et al., 2005) which are suppressed by dopaminergic treatments (Brown et al., 2001; Levy et al., 2002).

Changes in neural oscillations are associated with symptoms of PD. One of the prominent symptoms reflected in oscillatory activity is resting tremor. Significant coherence between tremor frequency (3–7 Hz) or its harmonic detected by EMG with the EEG or MEG signal from the contralateral hemisphere has been reported in several studies (Volkmann et al., 1996; Hellwig et al., 2000, 2001; Moran et al., 2008; Raethjen et al., 2009). Other studies have shown that the increase in subcortical oscillatory activity and intracortical coupling of neural activity are correlated with disease severity (Silberstein et al., 2005; Berendse and Stam, 2007; Stoffers et al., 2007; Stam, 2010).

In a recent study we found changes in cortico-striatal functional connectivity in PD patients using resting state functional connectivity MRI (fcMRI; Kwak et al., 2010). In PD patients we found an overall increase in the strength of corticostriatal functional connectivity, and L-DOPA alleviated these effects by decreasing connectivity strength. We interpreted these results as enhanced regional coupling of neural activation associated with the elevated synchronous neural oscillations in PD. However, it is not clear whether these changes in the resting state BOLD signal are confined to the corticostriatal circuitry. Although functional connectivity analysis can provide us with information on brain regions within particular networks, it does not reveal spontaneous BOLD signal changes across the whole brain. It is also unknown whether the resting state BOLD signal shows increased oscillations, parallel to what has been observed with EEG or MEG.

We addressed these issues in the current study by comparing the magnitude of resting state BOLD signal oscillations in PD patients ON and OFF L-DOPA and healthy age-matched controls using the amplitude of low frequency fluctuations (ALFFs). Developed by Zang et al. (2007), this approach involves the spectral decomposition of the time series data with a focus on amplitude in the low frequency domain relevant to the hemodynamic response function (i.e., below 0.08 Hz). The fractional ALFF (fALFF) is used as a normalized index of ALFF by providing the relative amplitude of the low frequency domain against the whole spectrum of frequencies (Zou et al., 2008). ALFF/fALFF gives the power of signal fluctuations, and thus indicates how much BOLD signal oscillation there is in a given region at rest. Because ALFF can be used to study the magnitude of the low frequency BOLD signal oscillations in a voxel-wise fashion across the whole brain, using this approach allows us to test the regional specificity of PD and L-DOPA effects on spontaneous BOLD signal oscillations.

Calculations of ALFF have recently gained much interest to study the dynamics of intrinsic brain activity (Yang et al., 2007; Biswal et al., 2010; Zuo et al., 2010). Furthermore, numerous studies have demonstrated changes in ALFF for neurological and psychiatric diseases (Zang et al., 2007; Hoptman et al., 2010; Huang et al., 2010; Liu et al., 2010, 2011; Lui et al., 2010; Zhou et al., 2010; Han et al., 2011). For example an increase in putamen ALFF has been reported in patients with paroxysmal kinesigenic choreoathetosis, which is a rare neurological condition where specific movements can induce dyskinesias (Zhou et al., 2010). For Schizophrenia, there have been reports of decreased ALFF in the medial prefrontal lobe and increased ALFF in the bilateral putamen (Huang et al., 2010). There have been two recent studies showing PD associated changes in ALFF (Skidmore et al., in press-a,b). These studies demonstrated that there are significant ALFF changes in a number of brain regions which reliably separate PD from healthy controls (Skidmore et al., in press-b). Furthermore, the ALFF measures correlated with apathy, depression and motor symptom severity in PD (Skidmore et al., in press-a). While these studies investigated disease effects, they did not evaluate changes in ALFF with anti-Parkinsonian medication.

Not many studies have approached ALFF with a clear hypothesis on the direction of change (i.e., whether a certain patient group will have higher or lower ALFF power) or what neural implications (i.e., whether it reflects signal oscillations at the neuronal level or whether it is just another index of resting state BOLD activity) ALFF power has. This is because it is a relatively newly developed measure. The current study extends efforts to understand the neural implications of ALFF by studying the PD population, in which abnormalities in neural oscillations have been identified. Based on prior neurophysiological investigations on PD, we hypothesized that there will be an increase in ALFF power for PD patients particularly in the corticostriatal network. More importantly we also investigated the effect of L-DOPA on ALFF measures, which allows us to determine whether the change in neural oscillatory activity by L-DOPA in PD is reflected in ALFF.

## Materials and methods

### Participants

The data set collected from 24 mild to moderate stage (Hoehn and Yahr stages 1–2.5; Hoehn and Yahr, 1967) PD patients (64 ± 8 years, two females) and 24 age- and gender-matched healthy controls (63 ± 7 years, five females) as part of a previous project (Kwak et al., 2010) was included in this study. Patients were evaluated using the motor section of the Unified Parkinson's Disease Rating Scale (UPDRS; Fahn et al., 1987). The more disease affected body side was determined by asking each PD patient and was confirmed by the UPDRS rating. All study participants underwent the Mini-Mental State Exam (MMSE; Folstein et al., 1975), the Montreal Cognitive Assessment (MOCA; Nasreddine et al., 2005) and the grooved pegboard test (Lafayette Instruments, Lafayette, IN) to measure general cognitive and psychomotor abilities. These clinical assessments were acquired for patients in both ON and OFF medication states. The demographic and clinical characteristics of the patients are listed in Table 1. Participants signed a consent form approved by the Institutional Review Board of the University of Michigan prior to the experiment, and were compensated for their participation. All experimental procedures were conducted in accordance with the Institutional Review Board of the University of Michigan.

**Table 1:** **Demographic and clinical variables of the Parkinson's patients**.

	**Age**	**M/F**	**Disease duration (years)**	**Affected body side**	**H&Y**	**UPDRS_ON**	**UPDRS_OFF**	**MMSE_ON**	**MMSE_OFF**	**MOCA_ON**	**MOCA_OFF**
PD_01	62	M	6	R	2.5	15	16	28	27	29	25
PD_02	70	M	8	L	2	23	19	28	28	27	30
PD_03	70	M	7	R	2	14	21	28	27	28	28
PD_04	52	M	6	R	2.5	16	20	30	30	26	30
PD_05	71	M	13	R	2.5	18	24	29	29	28	29
PD_06	69	M	12	R	2	20	24	30	30	30	27
PD_07	60	M	3	R	1.5	10	9	30	29	30	27
PD_08	65	M	8	R	2.5	21	27	29	30	27	28
PD_09	63	M	7	L	2.5	28	28	30	30	26	28
PD_10	69	M	9	R	2	24	23	29	29	22	22
PD_11	66	M	1	L	2	9	9	29	29	27	26
PD_12	67	M	9	R	2.5	14	14	27	27	27	28
PD_13	73	M	2	R	2.5	18	14	29	29	22	24
PD_14	51	M	2	R	2	9	8	30	29	30	27
PD_15	68	M	2	L	2	25	27	29	28	27	25
PD_16	72	M	4	L	2.5	27	26	27	28	21	25
PD_17	59	F	2	R	2	5	6	29	28	27	25
PD_18	64	M	7	R	2	8	6	28	29	18	20
PD_19	64	M	3	L	2	28	30	28	28	28	28
PD_20	80	M	6	R	2	11	13	27	28	29	27
PD_21	50	M	3	R	1.5	13	13	29	30	24	22
PD_22	62	M	2	R	2	10	17	29	28	26	25
PD_23	51	M	3	R	2.5	32	37	30	28	25	26
PD_24	55	F	3	R	2	15	14	29	30	22	24
Mean_PD	64.3 ± 8		5.4 ± 3		2.2 ± 0.3	17.3 ± 8	18.5 ± 8	28.8 ± 1	28.8 ± 1	26 ± 3	26 ± 3
Mean_Control	63.3 ± 7								29.1 ± 1		25.7 ± 3

### Procedure

PD patients completed two testing days corresponding to the ON and OFF medication states. Thirteen patients were tested ON first and 11 OFF first. Patients attended both testing days after withdrawal from medication for 12–18 h. Patients received a 50 mg dose of carbidopa followed by either a single dose of L-DOPA (ON) or placebo (OFF) in combination with 50 mg carbidopa (200 mg of L-DOPA/placebo and an additional 50 mg of carbidopa). All study procedures began 1 h after the patient had taken either L-DOPA or the placebo, by which time L-DOPA reaches its peak plasma dose. Patients were first assessed with the UPDRS (10 min), after which the resting fMRI scan was done (8 min) followed by an additional fMRI session not discussed in this paper (20 min). All other behavioral assessments including MMSE, MOCA and the grooved pegboard were done afterwards (10 min). Control participants underwent a single testing session without any medication procedure.

### fMRI data acquisition

FMRI data were collected from a 3T GE Signa MRI scanner. We acquired 240 T2^*^ - weighted BOLD images (TR = 2 s, TE = 30 ms, flip angle = 90°, FOV = 220 × 220 mm, voxel size = 3.4 × 3.4 × 3.2 mm, 40 axial slices) for 8 min during rest. A 3D T1 axial overlay (TR = 8.9 ms, TE = 1.8 ms, flip angle = 15°, FOV = 260 × 260 mm, slice thickness = 1.4 mm, 124 slices; matrix = 256 × 160) and T1-weighted anatomical image using spoiled gradient-recalled acquisition (SPGR) imaging (flip angle=15°, FOV = 260 × 260 mm, 1.4 mm slice thickness) were acquired. A pressure belt was placed around the abdomen to monitor the respiratory signal. A pulse oximeter was placed on the finger to monitor the cardiac signal. We did not separately monitor tremor during the fMRI scan.

### fMRI data analysis

The resting state fMRI data were preprocessed as follows: (1) *k-space* outliers were replaced with the average of their temporal neighbors, (2) images were reconstructed using field map correction, (3) physiological variations in the data from the cardiac and respiratory rhythms were regressed out, (4) images were slice timing and motion corrected. The preprocessed data were then normalized to MNI space using SPM5 (Wellcome Department of Cognitive Neurology, London, UK; http://www.fil.ion.ucl.ac.uk). In order to have all of the PD patients' predominantly disease affected hemisphere aligned, we flipped the *x* direction (i.e., left–right direction) of both the 240 functional images and the T1 anatomical images for the six left side more affected patients. The results are presented with the left side of the images reflecting patients' more affected brain hemisphere. The same proportion of control subject images was also flipped in the *x* direction. Functional images were spatially smoothed using a full width at half-maximum 8 mm Gaussian smoothing kernel.

The amplitude of spontaneous low-frequency fluctuations within the resting state fMRI time course was examined with ALFF and fALFF. For the purpose of conciseness, only the results of analyses conducted with ALFF are presented here. The fALFF results are presented in the Appendix. The time course data in each voxel was transformed into the frequency domain using fast-Fourier transformation. For ALFF, the sum of amplitudes within a specific low-frequency range (0–0.08 Hz) was computed at each voxel. fALFF, which is the ratio of the amplitude in a low-frequency band to amplitude in the total frequency band was computed by dividing the ALFF value by the total sum of amplitudes across the entire frequency range. ALFF and fALFF maps were *Z*-transformed by subtracting the mean voxel-wise ALFF or fALFF for the individual's entire brain, and dividing by the corresponding standard deviation. The individual *Z*-transformed ALFF and fALFF maps were carried forward to the group-level random effects analyses conducted with SPM5.

To determine L-DOPA effects on ALFF/fALFF in PD patients, we performed our analysis in the following fashion. First we compared PD OFF ALFF/fALFF maps to controls and to PD ON within anatomically defined ROIs comprised of the cortical motor areas, the prefrontal cortex (PFC), and the striatum. The PD ON data were also compared to controls to determine whether L-DOPA normalizes the altered resting state oscillations. A combination of M1, SMA, pre-SMA, and the dorsal and ventral premotor areas from the human motor atlas (Mayka et al., 2006) was used as the motor cortical ROI. A combination of the superior, middle, inferior and medial frontal gyri from the WFU PickAtlas software (Maldjian et al., 2003) was used as the PFC ROI. The caudate and putamen from the WFU PickAtlas software (Maldjian et al., 2003) were used as the striatal ROI. Each ROI encompassed both the left and right hemispheres. ROIs were used in the between group comparison using *p* < 0.005, uncorrected with an extent threshold of 20 voxels. Bonferroni corrections for multiple ROI comparisons were applied (0.005/3 ROIs), yielding a corrected threshold of *p* < 0.0017.

To determine whether L-DOPA normalizes PD associated changes, we also compared the ALFF/fALFF amplitudes between PD OFF versus PD ON and PD ON versus controls in the brain regions identified from the PD OFF versus control group comparison. The mean ALFF/fALFF amplitude across all voxels within the identified clusters was compared between PD OFF and PD ON and between PD ON and controls using repeated measures ANOVA. Medication status (i.e., PD OFF vs. PD ON) and ROI were used as within subject factors for the ON and OFF comparison and group (i.e., PD ON vs. controls) was used as a between subject factor and ROI as a within subject factor in the second comparison. In cases where the sphericity assumption was not met, the F statistic was evaluated for significance using the Huynh–Feldt adjusted degrees of freedom (Huynh and Feldt, 1970).

To determine whether PD-associated changes in ALFF/fALFF amplitudes have clinical implications, we explored correlations between the PD OFF ALFF/fALFF power of the regions identified from the PD OFF versus control group comparison and the clinical assessments. Mean ALFF/fALFF amplitude across all voxels within the identified cluster in PD OFF was used in this correlation analysis.

Lastly, exploratory whole brain comparisons of ALFF/fALFF maps were performed between the three groups using uncorrected *p* < 0.001 with an extent threshold of 20 voxels.

## Results

### Behavioral data group comparisons

There were no statistically significant age differences between PD patients and controls. Patients' performance on MMSE, MOCA and grooved pegboard tests ON and OFF L-DOPA states were compared to controls. Neuropsychological assessments were not performed for one control participant. In patients both ON and OFF L-DOPA performance on MMSE and MOCA did not differ from controls. Patients' grooved pegboard performance was worse than controls in both ON and OFF states for both the right and left hands (controls vs. PD ON: [*t*_(31.60)_ = 3.34, *p* < 0.005], right hand, [*t*_(27.10)_ = 4.3, *p* < 0.001], left hand, controls vs. PD OFF: [*t*_(29.49)_ = 3.9, *p* < 0.005], right hand, [*t*_(26.77)_ = 4.47, *p* < 0.001], left hand). We also compared patients' performance on the UPDRS, MMSE, MOCA, and grooved pegboard tests between the ON and OFF L-DOPA states using paired *t*-tests. No significant differences between medication states were found for MMSE or MOCA scores. Pegboard performance was analyzed separately for the more and less affected sides. Motor symptoms measured by UPDRS only showed a trend to be worse in PD OFF than PD ON [*t*_(23)_ = −2.0, *p* = 0.055], and pegboard performance for the more affected side was significantly worse for PD OFF than PD ON [*t*_(23)_ = −2.61, *p* < 0.05] but not for the less affected side. These results indicate that the patient group was comparable to controls in their general cognitive functions as shown by no significant group difference in MMSE and MOCA. However the results indicate that patients were impaired in manual motor performance compared to controls as shown by the difference in pegboard performance. Additionally the comparisons between PD ON and PD OFF indicate that L-DOPA significantly improved motor functioning of the patients, and the improvement was most apparent for the more affected side.

### Comparison of ALFF across groups: ROI analysis

**Figure 1:**
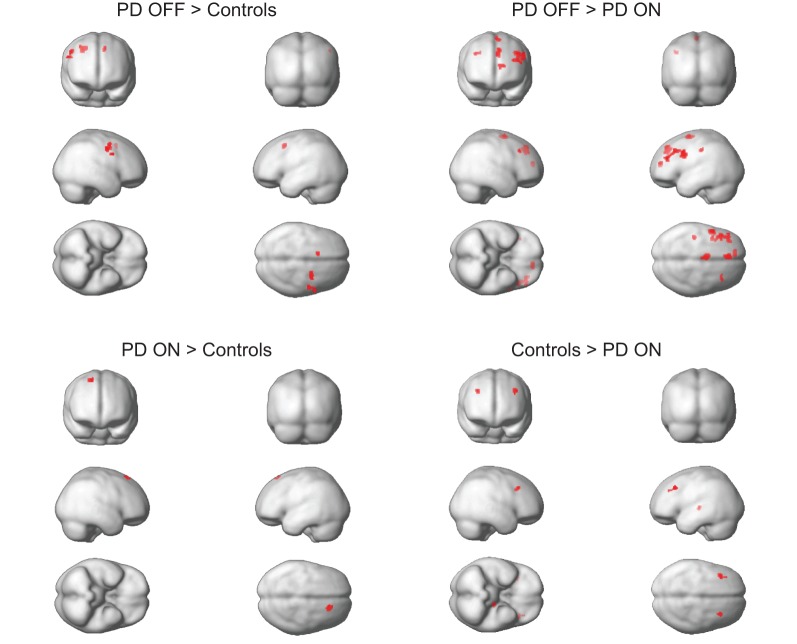
**Comparison of ALFF between groups within the predefined ROIs of the cortico-striatal network.** No significant results were found for controls > PD OFF or PD ON > PD OFF contrasts. *P* < 0.0017 based on correction.

We first compared the ALFF map of PD OFF to controls. We found that there was an overall increase in ALFF power in PD OFF compared to controls. In particular, ALFF power was increased in the primary and secondary motor areas (BA 6), the middle frontal gyrus (BA 8) of the less affected hemisphere and the medial frontal gyrus (BA 32) of the more affected hemisphere (Figure 1 and Table 2). There were no regions showing greater ALFF power in controls than PD OFF.

**Table 2:** **Brain regions where group differences in ALFF were found using the ROI analysis**.

	**Side (M/L)**	**BA**	**Coordinates**			***T* value**
			***x***	***y***	***z***	
**OFF > CONTROLS**						
Precentral gyrus	L	6	52	−4	42	4.28
Precentral gyrus (premotor)	L	6	52	4	32	3.61
Middle frontal gyrus	L	8	30	0	46	3.94
Medial frontal gyrus (ACC)	M	32	−10	14	46	3.92
**OFF > ON**						
Precentral gyrus (premotor)	M	6	−52	6	30	5.28
Medial frontal gyrus (SMA)	M	6	−6	−2	64	4.64
Middle frontal gyrus	M	8	−44	24	40	4.67
Middle frontal gyrus	L	8	36	26	38	3.61
Medial frontal gyrus	M	8	−4	38	38	4.28
Medial frontal gyrus	M	10	−10	52	16	3.82
**ON > CONTROLS**						
Superior frontal gyrus (pre-SMA)	L	6	20	28	62	3.99
**CONTROLS > ON**						
Thalamus (VPM)	M		−16	−22	2	4.08
Middle frontal gyrus	L	8	32	24	38	4.06
Middle frontal gyrus	M	8	−36	24	40	3.58

M/L refers to more and less affected sides, BA to Brodmann's area.

We then compared the ALFF map of PD OFF to PD ON and found that L-DOPA significantly reduced ALFF power in PD patients. These effects were most prominent in the prefrontal (BA 8 and 10) and motor cortical areas (BA 6; Figure 1 and Table 2) and were more evident in the more affected hemisphere. There were no regions showing L-DOPA associated increases in power.

We also compared PD ON to controls to determine whether L-DOPA normalizes PD-associated changes in low frequency oscillations. We found that some regions exhibited increased ALFF power and others decreases for PD ON compared to controls. Whereas the pre-supplementary motor area of the less affected hemisphere showed increases in PD ON, the thalamus of the more affected hemisphere and the middle frontal gyrus of both hemispheres (BA 8) showed decreases in ALFF power for PD ON compared to controls (Figure 1 and Table 2).

**Figure 2:**
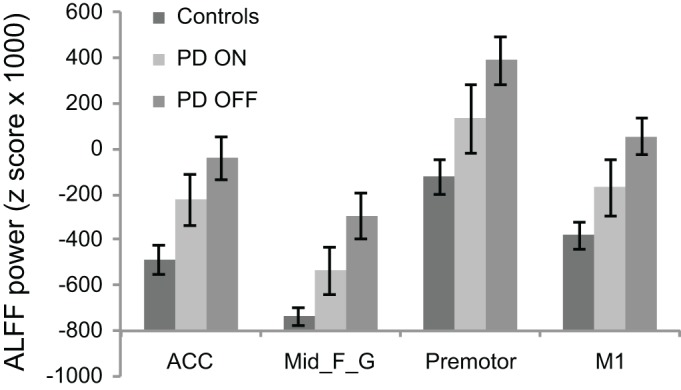
**ALFF power comparison of PD ON to PD OFF and PD ON to controls for brain regions identified in PD OFF > controls contrast.** L-DOPA significantly down-regulates the abnormally increased ALFF power in PD as shown by the PD ON vs. PD OFF comparison. A difference in ALFF power still existed after L-DOPA administration as shown by PD ON vs. controls comparison. The negative *y* axis values (ALFF power *z* score) indicate ALFF power lower than the mean of the whole brain. ACC, anterior cingulate cortex; Mid_F_G, middle frontal gyrus; M1, precentral gyrus.

To determine whether the PD-associated increase in ALFF power is down-regulated by L-DOPA, we performed an additional ROI analysis comparing the ALFF power between PD OFF and PD ON and between PD ON and controls within the regions that were identified as exhibiting greater ALFF for PD OFF relative to controls. A comparison between PD OFF and PD ON using a medication status (i.e., PD OFF vs. PD ON) by ROI (i.e., precentral vs. premotor vs. mid_frontal_g vs. ACC, see Table 2. PD OFF > Controls for clusters used as ROIs) repeated measures ANOVA showed a main effect of medication status showing overall decreases in average ALFF power across the four ROIs [*F*_(1, 23)_ = 6.29, *p* < 0.05, Figure 2]. There was also a main effect of ROI [*F*_(2.02, 46.40)_ = 14.94, *p* < 0.001]. No significant interaction was found. A comparison between PD ON and controls using a group (i.e., PD ON vs. control) by ROI repeated measures ANOVA also showed a main effect of group [*F*_(1, 46)_ = 5.17, *p* < 0.05] and a main effect of ROI (*F*_(3, 138)_ = 23.76, *p* < 0.001). No significant interaction was found.

### ALFF correlations with behavioral metrics in PD

**Figure 3:**
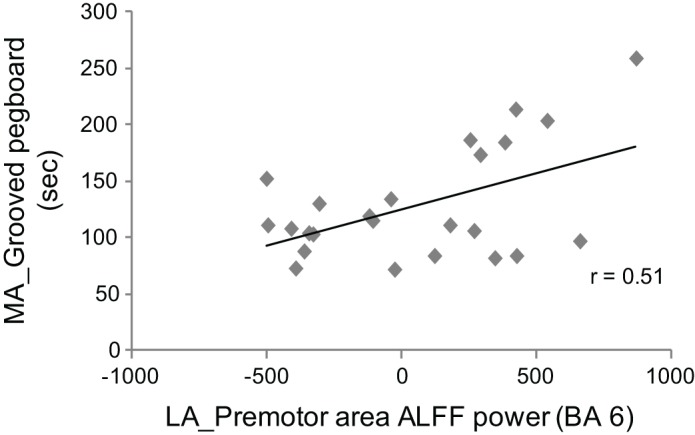
**Correlation between ALFF of the premotor cortex in the less affected hemisphere (LA) and performance on the grooved pegboard task of the more affected body side (MA) in PD OFF.** Greater ALFF is associated with worse performance (i.e., slower movement).

We explored correlations between the PD-related changes in ALFF power (regions showing a difference between PD OFF and controls listed in Table 2) with grooved pegboard based on the observation that performance differed between patients and controls. The UPDRS score was also used in the correlation analysis considering its significance in disease rating. Mean ALFF power across all voxels for each of the four cluster regions and the clinical variables from patients in the OFF state were used. We only found correlation between the ALFF power of the premotor cortex in the less affected hemisphere and the more affected hand performance on the grooved pegboard test. Poorer performance was associated with higher ALFF power (*r* = 0.51, *p* = 0.012, Figure 3).

### Comparison of ALFF across groups: whole brain analysis

**Figure 4:**
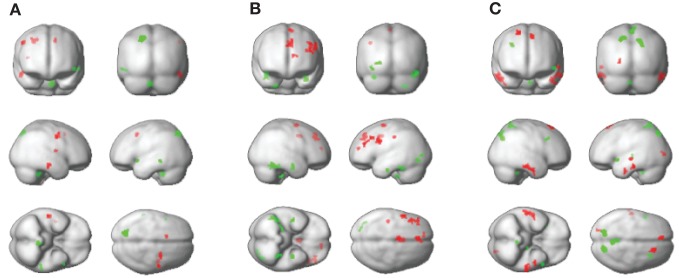
**Whole brain ALFF comparisons between groups.** PD OFF > controls, red, PD OFF < controls, green **(A)**, PD OFF > PD ON, red, PD OFF < PD ON, green **(B)**, PD ON > controls, red, PD ON < controls, green **(C)**.

A comparison of whole brain ALFF maps between PD OFF and controls showed elevated power in PD OFF predominantly in the primary and secondary motor areas (BA 6), which was already identified in the ROI analysis (Figure 4A and Table 3). The only other brain region that showed greater power in PD OFF was the inferior temporal gyrus. ALFF power was decreased in PD OFF in the precuneus, cerebellum lobule V, and superior and middle temporal gyri (Figure 4A and Table 3).

**Table 3:** **Brain regions where group differences in ALFF were found with whole brain analysis**.

	**Side (M/L)**	**BA**	**Coordinates**			***T* value**
			***x***	***y***	***z***	
**OFF > CONTROLS**						
Precentral gyrus	L	6	42	−6	14	4.4
Inferior temporal gyrus	L	20	54	−20	−18	4.2
Middle frontal gyrus (SMA)	L	6	30	0	46	3.94
Medial frontal gyrus (pre-SMA)	M	6	−10	14	46	3.92
**CONTROLS > OFF**						
Precuneus	M	7	−20	−74	52	4.6
Cerebellum (lobule V)	M		−6	−42	−36	4.18
Superior temporal gyrus	M	38	−48	12	−8	4.12
Middle temporal gyrus	M	21	−58	−36	−8	3.73
**OFF > ON**						
Inferior frontal gyrus	M	44	−52	6	30	5.28
Medial frontal gyrus (ACC)	M	32	−2	8	42	5.06
Medial frontal gyrus	M	8	−4	38	38	4.28
Postcentral gyrus	M	1/2	−40	−26	40	4.74
Middle frontal gyrus	M	9	−44	24	40	4.67
Superior frontal gyrus (SMA)	M	6	−6	−2	64	4.64
Superior frontal gyrus	M	10	−10	52	16	3.82
**ON > OFF**						
Inferior temporal gyrus	M	20	−36	−10	−30	6.44
Cerebellum (lobule V)	M		−34	−42	−28	5.25
Cerebellum (lobule X)	L		26	−32	−44	4.64
Fusiform gyrus	L	37	50	−54	−16	4.41
Middle temporal gyrus	L	21	42	−10	−20	4.27
Occipital gyrus	M	18	−22	−86	2	4.21
**ON > CONTROLS**						
Inferior temporal gyrus	L	20	56	−10	−32	4.55
Inferior temporal gyrus	M	20	−46	−6	−30	4.07
Middle temporal gyrus	M	21	−58	−18	−6	4.26
Superior frontal gyrus	M	8	−6	48	54	4.18
Superior frontal gyrus (premotor)	L	6	20	26	64	4.09
Fusiform gyrus	M	20	−64	−24	−24	3.97
Cuneus	M	17	−22	−86	8	3.92
**CONTROLS > ON**						
Superior occipital gyrus	L	19	20	−76	38	4.42
Superior temporal gyrus	M	12	−50	10	−8	4.31
Thalamus (VPM)	M		−16	−22	2	4.08
Middle frontal gyrus	L	9	32	24	38	4.06
Precuneus	M	7	−14	−74	52	4.04
Precuneus	L	7	2	−58	64	3.88

M/L refers to more and less affected sides, BA is Brodmann's area.

A comparison of whole brain ALFF maps between PD OFF and PD ON showed decreased power in PD ON in multiple regions within the PFC (BA 6, 8, 9, 10, 32, and 44) that were already identified in the ROI analysis, as well as in the postcentral gyrus (BA 1/2; Figure 4B and Table 3). ALFF power was increased in PD ON compared to PD OFF in the middle and inferior temporal gyri, the occipital and fusiform gyri and cerebellum lobules V and X (Figure 4B and Table 3). A comparison of PD ON to controls showed areas of increased ALFF power including the middle and inferior temporal gyri, superior frontal (BA 6, 8) and fusiform gyri and the cuneus (Figure 4C and Table 3) as well as decreased power in the superior occipital, superior temporal, middle frontal gyrus, thalamus and precuneus (Figure 4C and Table 3).

**Figure 5:**
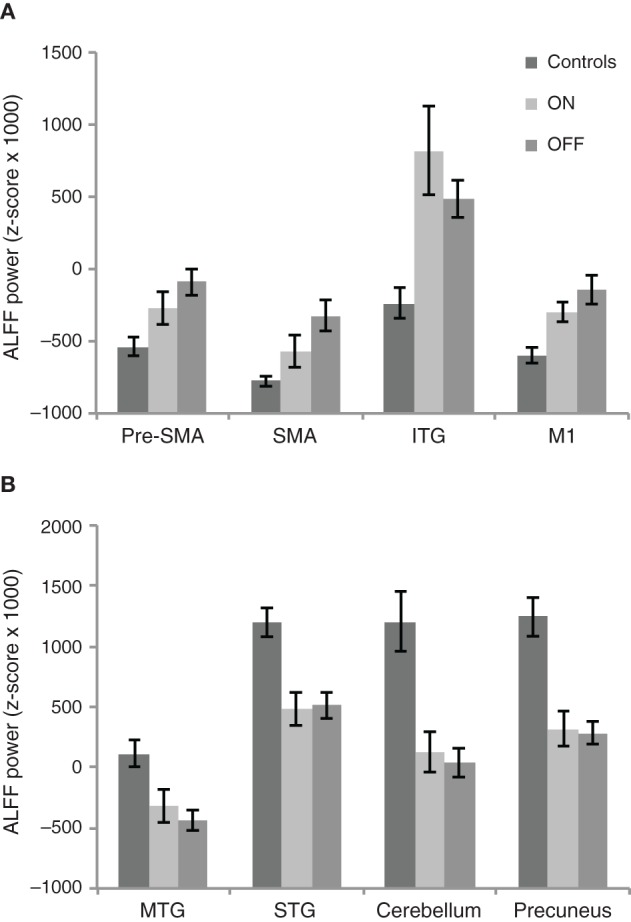
**ALFF power of the three groups (i.e., PD ON, PD OFF, and controls) for brain regions identified in PD OFF > controls contrast (A) and in PD OFF < controls contrast (B) in the whole brain analysis**.

We also performed ROI analyses comparing the ALFF power between PD OFF and PD ON and between PD ON and controls within the regions that were identified as exhibiting differences between PD OFF relative to controls in the whole brain. Two separate analyses were done for ROIs identified as showing greater ALFF power in PD OFF than controls and for ROIs identified as showing lower ALFF power in PD OFF than controls. In the former, we used average ALFF power across the whole cluster of pre-SMA, SMA, precentral gyrus and the inferior temporal gyrus (ROI set 1, Figure 5A). In the latter we used the average ALFF power of the precuneus, cerebellum, middle, and superior temporal gyri (ROI set 2, Figure 5B, see Table 3 for the peak voxel coordinates of the ROI clusters used). A repeated measures ANOVA comparing PD OFF and PD ON across ROI set 1 showed a main effect of ROI [*F*_(3, 69)_ = 18.4, *p* < 0.0001] and a marginal group by ROI interaction effect [*F*_(3, 69)_ = 2.7, *p* = 0.05]. A follow up paired *t*-test comparing each ROI between groups did not show group differences in any of the ROIs. A repeated measures ANOVA comparing PD OFF and PD ON across ROI set 2 only showed a ROI main effect [*F*_(3, 69)_ = 12.9, *p* < 0.0001]. A repeated measures ANOVA comparing PD ON and controls across ROI set 1 showed a significant main effect of group [*F*_(1, 46)_ = 18.4, *p* < 0.0001], ROI [*F*_(1.5, 68.8)_ = 20.8, *p* < 0.0001], and a group by ROI interaction [*F*_(1.5, 68.8)_ = 4.8, *p* < 0.05]. A follow up two sample *t*-test comparing each ROI between groups showed that while group differences were found for the pre-SMA [*t*_(46)_ = −2, *p* < 0.05], precentral [*t*_(46)_ = −3.5, *p* < 0.005] and inferior temporal gyrus [*t*_(28)_ = −3.2, *p* < 0.005], no group difference was found for the SMA. A repeated measures ANOVA comparing PD ON and controls across ROI set 2 showed a main effect of group [*F*_(1, 46)_ = 60.5, *p* < 0.0001] and ROI [*F*_(3, 138)_ = 14.4, *p* < 0.0001], and no group by ROI interaction.

## Discussion

In the current study, we determined whether L-DOPA changes the resting state low frequency BOLD signal oscillations in PD patients. We hypothesized that L-DOPA would reduce the abnormality of signal oscillations in PD patients. We found that there was an abnormal elevation of ALFF power in PD specifically within the primary and secondary motor regions and the PFC. Individual differences in the ALFF measure predicted clinical motor symptoms measured by Grooved Pegboard. This task has been previously shown to be a sensitive index of the level of nigrostriatal dopaminergic denervation in PD (Bohnen et al., 2007). We found that the ALFF power in the premotor cortex within PD patients explains some of the variance in pegboard performance, which reflects striatal dopaminergic denervation and thus, disease severity. That is, the greater the ALFF amplitude, the worse the pegboard performance and thus symptom severity. It is of note that this relationship was found between the less affected hemisphere and the more affected hand performance, which suggests that the correlation we observed may not reflect direct functional coupling between ALFF power and pegboard performance. Furthermore, considering that the correlation did not survive multiple comparison correction the result should be interpreted with caution.

In our comparisons between PD OFF and PD ON and PD ON and controls, we showed that L-DOPA significantly alleviated the increased ALFF power in the primary and secondary motor areas and the prefrontal regions. We also confirmed that L-DOPA decreased ALFF power within the brain regions showing increases in PD compared to controls, although there were still significant differences from controls. The L-DOPA associated ALFF changes revealed by the comparison of PD ON and PD OFF were more prominent in the more disease-affected hemisphere. L-DOPA has been shown to selectively increase synaptic dopamine in the more denervated subregions of the striatum (Tedroff et al., 1996). Additionally whereas the L-DOPA associated synaptic dopamine increase is significant versus baseline in the more affected putamen, it was not in the less affected side (Tedroff et al., 1996). It is possible that the controlled dose of L-DOPA we used primarily affected the more denervated hemisphere, contralateral to most Parkinsonian symptoms thereby changing the ALFF amplitude to a greater extent in the more affected hemisphere. Collectively these results suggest that L-DOPA modulates ALFF power, such that it down-regulates the abnormally increased oscillation in the BOLD signal in PD.

It is of note that the L-DOPA dosage we used did not effectively alleviate motor symptoms as assessed by UPDRS, although there was a trend and also a significant improvement in the Pegboard performance. UPDRS was assessed after 1 h from either L-DOPA or placebo administration, which is when the L-DOPA reaches its peak plasma dose and its effect reaches the peak. Thus it is not likely that the assessment was done after the L-DOPA effect subsided. It is possible that the dosage we used may not have been effective for all PD patients, since some of them were usually taking both L-DOPA and dopamine agonists or only agonists. It is important to note that the changes in ALFF with L-DOPA are found even when L-DOPA effects on symptom improvement are not significant. This suggests that the neural effects we see in ALFF could be capturing changes in the brain state prior to symptomatic change.

Comparisons of ALFF maps between PD ON and controls showed patterns of both increased and decreased power in PD ON. It is possible that depending on the efficacy of the controlled dose of L-DOPA for our patient group, it does not sufficiently decrease the abnormal oscillations in some regions. This is also supported by the still existing gap in ALFF power between controls and patients even after L-DOPA administration. The results also demonstrate that L-DOPA suppresses the oscillations even beyond the normal range in some regions.

Although we found significant group differences in the primary and secondary motor areas and the prefrontal regions, which are the major cortical target of striatal output, we found little effect in the striatum. The only striatal region we found was in the caudate where fALFF power was increased in PD OFF compared to PD ON (see Appendix). A previous study has shown that cortical regions show relatively higher f/ALFF power than subcortical regions in the healthy brain (Zuo et al., 2010). We speculate that group differences are better identified in cortical than in subcortical regions because there is room for a wider range of effects.

Our findings differ from those of a recent study comparing ALFF between PD patients and controls (Skidmore et al., in press-b). While this study did not test patients in the ON medication state, the results showed that ALFF power was *decreased* in patients relative to controls in a number of regions including the supplementary motor cortex, the mesial PFC, and the middle frontal gyrus (Skidmore et al., in press-b). The only brain region that showed increased ALFF power in PD was the right cerebellum. There are a number of methodological differences between our study and theirs', which may have led to the discrepancies. First, we evaluated data from 24 patients while Skidmore et al. (in press-a,b) included 15. Additionally in our study the more and less disease affected hemispheres were aligned across patients before statistical analysis as in our previous study (Kwak et al., 2010). Furthermore, we acquired respiratory and cardiac signals during scanning and regressed these signals out of the resting state fMRI data, while Skidmore et al. (in press-a,b) regressed out the full brain global mean signal. The latter has been shown to dramatically shift the profile of resting state brain correlation patterns (Murphy et al., 2009; Van Dijk et al., 2010) and may have contributed to differences in the pattern of findings between the two studies.

The current findings are in line with those of our recent investigation comparing the strength of cortico-striatal functional connectivity between PD patients ON and OFF L-DOPA and age-matched controls (Kwak et al., 2010). The results also parallel the findings of a recent fMRI study showing increased resting state functional connectivity between the subthalamic nucleus and cortical motor areas (BA 4/6) in PD (Baudrexel et al., 2011). The enhanced low frequency BOLD signal oscillations in the motor and prefrontal regions may underlie the increased functional connectivity shown in these previous studies (Kwak et al., 2010; Baudrexel et al., 2011), which parallels findings shown by others using local field potential (LFP) and EEG/MEG recordings in PD (Schnitzler and Gross, 2005; Gatev et al., 2006; Berendse and Stam, 2007; Hammond et al., 2007; Timmermann and Fink, 2009; Stam, 2010). However, there is not an obligatory link between increased resting state BOLD oscillations and functional connectivity; the latter would require an increase in *synchronicity* of the BOLD signal across multiple regions.

The current work is closely related to our prior publication using seed-based functional connectivity analysis (Kwak et al., 2010). However the approach we take in the current study is distinctive from our previous study in the following ways: (1) While in the previous study we looked at the degree of synchronous activation between striatal seed region time courses and voxel time courses of the rest of the brain, in the current study we focused on the intrinsic oscillations within a brain region. (2) With the seed-based functional connectivity used in the previous study, we could only look at changes in predetermined sets of networks (i.e., striatal networks) whereas in the current study we looked at changes across the whole brain by a voxel-wise comparison of ALFF power of the whole brain. It should be stressed that correlation-based functional connectivity and power frequency measurements give different information about the low-frequency time courses; there can be a change in power without a change in correlation, and *vice-versa*. Thus, the prior seed-based work does not speak to changes in ALFF power covered in the current work.

As suggested by prior studies, tremor can be reflected in neural oscillations (Volkmann et al., 1996; Hellwig et al., 2000, 2001; Moran et al., 2008; Raethjen et al., 2009) and could be linked to the changes we observe in ALFF in PD patients. A limitation of the current study is that we did not have an online measure of tremor during our scanning session. However, tremor was also not the most significant symptom in most of our patients. The range of UPDRS tremor scores across all of our subjects was 0–7 while the score could range between 0 and 32. Eighteen out of 24 patients had a score of 2 or lower. Thus based on the UPDRS tremor score, it is unlikely that tremor significantly affected the degree of ALFF. Moreover there was no significant difference between tremor score ON and OFF, whereas significant differences in ALFF were found in multiple regions of the brain. Head motion inside the scanner reflects movement, and thus may also capture tremor during the scan. We regressed out head motion prior to data analyses. In order to determine whether ALFF is explained by the degree of tremor measured by UPDRS, we explored correlations between ALFF power of the four cluster regions identified in the PD OFF vs. Controls comparison and the UPDRS tremor score in both the OFF and ON state. UPDRS tremor score did not correlate with ALFF power of any of the cluster regions for patients in either the OFF or ON state. However, considering that UDPRS tremor is not the most accurate measure of tremor and since we did not acquire an online time course of tremor during the scan, we cannot definitively conclude whether changes in ALFF reflect changes in neuronal oscillations associated with tremor.

Whether or not the changes we observed in the very low frequency oscillations of the BOLD signal is parallel to the changes in neural oscillations found in prior LFP and EEG/MEG studies in PD requires careful consideration. Because the BOLD signal is derived from the hemodynamic response, one source of BOLD signal oscillation that may not be neurally driven is that associated with the mechanical fluctuations of the cerebral vasculature. Studies have indeed found that BOLD signal oscillations may contain properties of vascular oscillation (Strik et al., 2002; Wise et al., 2004; Frederick et al., 2012). At the same time however, these studies do not demonstrate that BOLD signal fluctuations are purely driven by the physical properties of the vasculature. Even after removing the vasculature signals, BOLD signal fluctuations demonstrate neuronal properties as shown by the presence of previously identified functional networks. It is of note that we cannot exclude the possibility that differences in BOLD signal fluctuation between PD patients and controls or between L-DOPA states are primarily driven by differences in non-neuronal vascular oscillation as dopamine affects cerebral vascular properties (Gleason et al., 2002). Thus our results should be interpreted carefully considering the contribution of dopamine to cerebral vasculature. Future studies are warranted to address this issue by measuring BOLD and vascular properties independently in dopamine ON and OFF states.

It is important to note that the current study does not aim to identify the frequency range of BOLD oscillations with that of the signal from the microcircuit level. BOLD fMRI is a hemodynamic proxy for and an aggregate representation of the underlying neural activities, which may oscillate in different frequencies. Simultaneous intra-cortical recordings and fMRI have demonstrated that the BOLD signal correlates with different types of neural activity such as LFPs, multi-unit activity and single-unit activity in multiple different frequency ranges such as delta, theta, alpha, and gamma bands (Logothetis et al., 2001; Goense and Logothetis, 2008). Simultaneous EEG and fMRI studies have also shown that the EEG signal in these different frequency bands collectively contributes to BOLD oscillations (Moosmann et al., 2003; Laufs, 2008; de Munck et al., 2009; Britz et al., 2010; Musso et al., 2010; Rosa et al., 2010). Thus the actual frequency range for the BOLD signal (~0.08 Hz) may contain information from neural activity in multiple different ranges, and not confined to the frequency range we observed. To determine how neural signals from different frequency bands contribute to changes in BOLD signal oscillations associated with PD and L-DOPA, a combined EEG-resting state fMRI study in PD patients is warranted.

In summary, we found L-DOPA associated changes in the resting state low frequency BOLD signal oscillations within the prefrontal and motor cortices, which are major cortical output regions of the striatum. L-DOPA significantly reduced the PD-associated abnormality in BOLD signal oscillations. These results have significant implications for understanding pathological neural oscillations in PD. This study suggests that resting state BOLD signal oscillations may provide a useful means to study the pathophysiology of PD and the physiology of dopaminergic pathways in healthy individuals.

### Conflict of interest statement

The authors declare that the research was conducted in the absence of any commercial or financial relationships that could be construed as a potential conflict of interest.

## Figures and Tables

**Figure A1:**
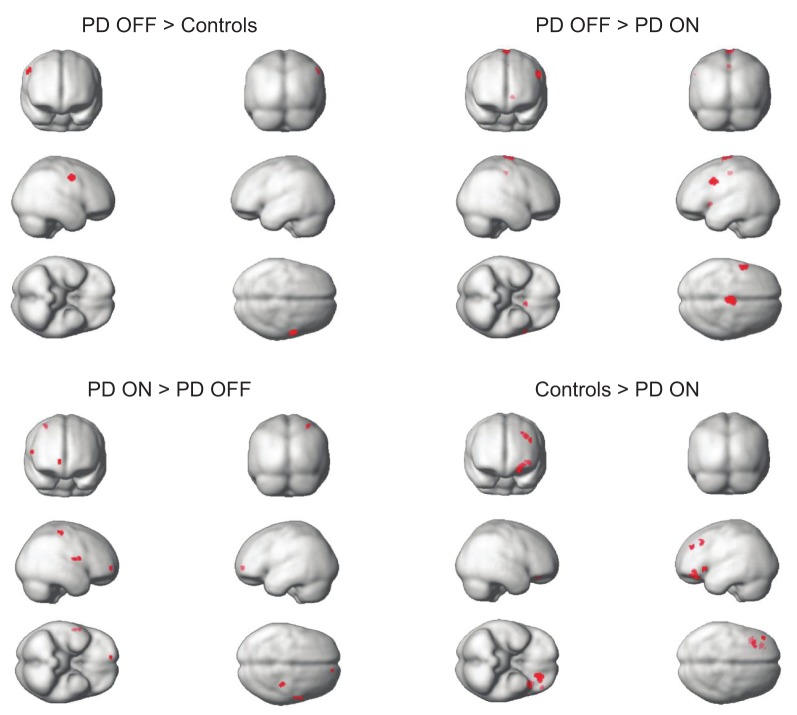
**Comparison of fALFF between groups within the predefined ROIs of the cortico-striatal network.** No significant results were found for controls > PD OFF or Controls > PD ON contrasts. *P* < 0.0017 based on correction.

**Figure A2:**
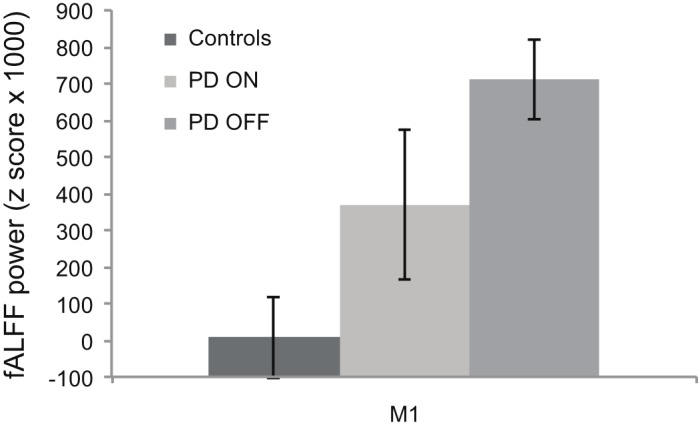
**fALFF power comparison of PD ON to PD OFF and PD ON to controls for brain regions identified in PD OFF > controls contrast.** L-DOPA down-regulates the abnormally increased fALFF power in PD as shown by the marginal difference in PD ON vs. PD OFF comparison. Difference in fALFF power was no more present after L-DOPA administration as shown by PD ON vs. controls comparison. M1, precentral gyrus.

**Figure A3:**
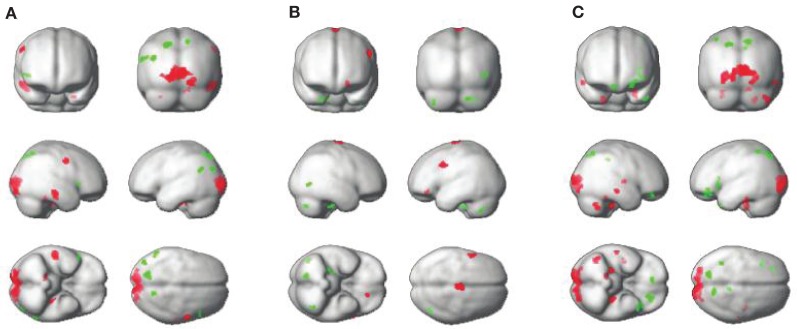
**Whole brain fALFF comparisons between groups.** PD OFF > controls, red, PD OFF < controls, green **(A)**, PD OFF > PD ON, red, PD OFF < PD ON, green **(B)**, PD ON > controls, red, PD ON < controls, green **(C)**.

## Appendix

### Comparison of fALFF across groups: ROI analysis

We first compared the fALFF map of PD OFF to controls. Similar to ALFF, we found increased fALFF power in PD OFF compared to controls and there were no regions showing greater BOLD signal power in controls than PD OFF. fALFF power was increased in the precentral gyrus of the less affected hemisphere (BA 6; Figure A1 and Table A1).

**Table A1:** **Brain regions where group differences in fALFF were found using the ROI analysis**.

	**Side (M/L)**	**BA**	**Coordinates**			***T* value**
			***x***	***y***	***z***	
**OFF > CONTROLS**						
Precentral gyrus	L	6	58	−4	42	4.31
**OFF > ON**						
Medial frontal gyrus (SMA)	L	6	2	−18	78	4.88
Middle frontal gyrus	M	9	−56	12	36	4.34
Caudate	M		−10	12	−2	4.29
**ON > OFF**						
Medial frontal gyrus	L	10	10	58	0	4.63
Precentral gyrus	L	4	34	−26	60	4.17
Precentral gyrus	L	4	58	4	16	3.7
**CONTROLS > ON**						
Inferior frontal gyus	M	11	−22	34	−20	4.17
Inferior frontal gyus	M	47	−36	20	−2	3.74
Middle frontal gyrus	M	8	−34	26	40	3.87
Superior frontal gyrus	M	9	−42	44	32	3.74

M/L refers to more and less affected sides, BA is Brodmann's area.

Comparison of fALFF maps between PD OFF and PD ON revealed some regions that showed decreased power but others that showed increased power with L-DOPA. Whereas the supplementary motor area (BA 6) of the less affected hemisphere and the dorsolateral PFC (BA 9) and caudate nucleus of the more affected hemisphere showed L-DOPA associated decreases in fALFF power, the medial prefrontal (BA 10) and precentral (BA 4) cortices of the less affected hemisphere showed L-DOPA associated increases in fALFF power (Figure A1 and Table A1).

Comparison of fALFF maps between PD ON and controls showed decreased fALFF power in the prefrontal regions (BA 8, 9, 11 and 47) of the more affected hemisphere in PD ON compared to controls (Figure A1 and Table A1). There were no regions showing increased fALFF power in PD ON versus controls.

Additional ROI analyses were performed to compare fALFF power between PD OFF and PD ON and between PD ON and controls within the regions that were identified as exhibiting greater fALFF for PD OFF relative to controls. A paired *t*-test comparing the average power of the precentral gyrus ROI between PD OFF and PD ON showed a marginally significant difference with decreased power in PD ON compared to PD OFF [*t*_(23)_ = 1.81, *p* = 0.08, Figure A2]. An independent *t*-test comparing PD ON and controls showed no significant difference in average fALFF power [*t*_(46)_ = −1.55, *p* > 0.1]. PD-related changes in fALFF power (relative to controls) did not show any correlations with clinical variables.

### Comparison of fALFF across groups: whole brain analysis

fALFF map whole brain comparisons between the three groups showed a similar pattern as the ALFF map comparisons (Figure A3 and Table A2).

**Table A2:** **Brain regions where group differences in fALFF were found with whole brain analysis**.

	**Side (M/L)**	**BA**	**Coordinates**			***T* value**
			***x***	***y***	***z***	
**OFF > CONTROLS**						
Cuneus	M	18	−8	−96	4	5.87
Inferior temporal gyrus	L	20	54	−24	−14	5.85
Lingual gyrus	L	18	18	−94	−12	5.64
Precentral gyrus	L	6	58	−4	42	4.31
Cerebellum (lobule III)	L		16	−40	−24	4.16
Cerebellum (lobule V)	M		−30	−30	−36	3.92
**CONTROLS > OFF**						
Precuneus	M	7	−16	−72	52	5.03
Superior occipital gyrus	M	19	−42	−82	30	4.53
Inferior parietal lobule	M	39	−60	−58	26	4.49
Superior parietal lobule	L	7	14	−64	56	4.47
Inferior frontal gyrus	M	47	48	20	0	3.9
**OFF > ON**						
Middle frontal gyrus	M	11	−18	38	−14	5.15
Middle frontal gyrus	M	9	−56	12	36	4.34
Medial frontal gyrus	L	6	2	−18	78	4.88
**ON > OFF**						
Inferior temporal gyrus	L	19	42	−70	0	5.11
Cerebellum (Cr I)	L		16	−74	−38	4.96
cerebellum (Cr II)	M		−40	−60	−46	4.86
Cerebellum (lobule V)	L		24	−28	−40	4.18
**ON > Controls**						
Cuneus	L	17	4	−94	6	5.94
Cerebellum (lobule X)	L		26	−30	−42	4.86
Cerebellum (Cr I)	L		40	−60	−36	4.7
Cerebellum (Cr V)	L		16	−42	−24	4.48
Middle temporal gyrus	L	21	58	−22	−12	4.46
Infereior temporal gyrus	M	20	−28	−26	−34	4.34
Insula	L	13	40	−6	6	3.98
**CONTROLS > ON**						
Superior parietal lobule	L	7	14	−64	54	4.59
Superior parietal lobule	M	7	−38	−50	60	4.02
Precuneus	M	7	−20	−76	50	4.4
Precuneus	L	7	10	−36	44	3.99
Superior temporal gyrus	M	38	−48	18	−38	4.37
Orbital gyrus	L	11	8	42	−20	4.17
Middle frontal gyrus	M	11	−22	34	−20	4.17
Inferior frontal gyrus	M	47	−36	20	−2	3.74

M/L refers to more and less affected sides, BA is Brodmann's area.
